# Assessment of the lethal and sublethal effects by spinetoram on cotton bollworm

**DOI:** 10.1371/journal.pone.0204154

**Published:** 2018-09-14

**Authors:** Jizhen Wei, Lili Zhang, Shuo Yang, Bingtang Xie, Shiheng An, Gemei Liang

**Affiliations:** 1 State key Laboratory of Wheat and Maize Crop Science/College of Plant Protection, Henan Agricultural University, Zhengzhou, China; 2 State Key Laboratory for Biology of Plant Diseases and Insect Pests, Institute of Plant Protection, Chinese Academy of Agricultural Sciences, Beijing, China; Institute of Plant Physiology and Ecology Shanghai Institutes for Biological Sciences, CHINA

## Abstract

*Helicoverpa armigera* is an universal pest around the world, which has recovered again in recent years because of the adjustment of cropping structure and resistance to *Bacillus thuringiensis* (Bt) in China. As a new insecticide spinetoram is extensively used to control many pest insects, including *H*. *armigera*. However the lethal and sublethal effects of spinetoram on cotton bollworm have not been assessed. In the present study, the toxicity of spinetoram against cotton bollworm was tested under laboratory conditions. Results demonstrated spinetoram showed an excellent activity against *H*. *armigera*, especially, against Bt (Cry1Ac) resistant *H*. *armigera*. Treatment with spinetoram at the doses of 0.19 mg/kg and 0.36 mg/kg (LC_8_ and LC_20_ after 24h oral exposure) significantly arrested the development of surviving larvae and caused significant decrease in larvae wet weight. Besides, the survivors after spinetoram treatments showed significant reduction of pupation ratio, pupal weight, emergence ratio, longevity and fecundity of adults. At same time, spinetoram treatments resulted in significant increase in the prepupal and pupal periods of survivors. In summary, these results showed that spinetoram could be used as an effective pesticide to control *H*. *armigera*, especially Cry1Ac-ressitacne, consequently to take both lethal and sublethal effects to cotton bollworm into consideration in cotton bollworm control strategy.

## Introduction

*Helicoverpa armigera* is one of the most damaging agricultural pests in the tropical and subtropical areas of the world. In northern China, cotton bollworm occured about four generations a year. In general, the first-generation cotton bollworm larvae were always found in wheat field, and the subsequent generation transfered to cotton, corn, peanuts, soybeans, vegetables and so on [1]. In China, the long-distance migrations of cotton bollworm between provinces and dispersal among different host crops led to successive, economically significant outbreaks of this pest during the early 1990s [2]. For controlling cotton bollworm, *Bacillus thuringiensis* (Bt) *Cry1Ac* gene was genetically modified to cotton in 1997 in China [3]. By 2017, Bt cotton had been extensively planted at a rate of nearly 100% in Eastern and Northern China, which effectively decreased the use of insecticides, increased natural enemies and increased yields [4–7]. Although *H*. *armigera* populations began to decline markedly by around 2000, the species has recovered again since 2010 in China [4, 8]. The adjustment of cropping structures answered for recovery of this pest, which led to a change in the pest status of the cotton bollworm. More farmers tended to plant corn, vegetable, peanuts, etc in Northern China, instead of cotton [4, 8]. In addition, the outbreak of cotton bollworm partly caused by its ability to become resistance to chemical pesticides [9]. Owing to the heavy use of carbamate, organochlorine, pyrethroid insecticides, and organophosphate, including Bt over the past six decades, cotton bollworm has exhibited resistance to all these insecticides [10–13]. For example, field populations of cotton bollworm have shown significant resistance to Cry1Ac after nearly two decades of feeding on transgenic cotton plants producing Cry1Ac in China [14–16]. The resistance frequency to Bt has regularly and dramatically increased in several pests and has reduced the benefits [13, 17].

In order to control cotton bollworm effectively and increase agricultural productivity continuously, many insecticides, which have the novel modes of action, have been introduced, including spinetoram. Spinetoram and spinosad are both in the class of spinosyn insecticides. Spinosad is naturally extracted from the fermentation product of *Saccharopolyspora spinosa*, including spinosyn A and spinosyn D, two macrocyclic lactones [18]. Different to spinosad, spinetoram is that originated from the fermentation product of *S*. *spinosa* with chemical modification spinetoram, consists of two active ingredients, XDE-175-J and XDE-175-L with the ratio about three to one [19]. Spinosyns was reported to activate nicotinic acetylcholine receptor (nAChR), specifically the Dα6 subunit, therefore to destroy the normal function of GABA-gated chloride channels, caused the overexcitement of the insect nervous system, finally result in paralysis and insect death [20, 21]. Although both spinetoram and spinosad act on the insect nervous system, their mode of actions may be different based on the different active ingredients. Unfortunately, as a new insecticide, the information concerning spinetoram receptors was largely unknown.

Since the broad-spectrum activity that provides long-lasting control of insect pests (Hymenoptera, Diptera, Siphonaptera, Lepidoptera and Thysanoptera) in a variety of crops [22–27], the high effectiveness of spinetoram to rice pests [28], noctuidae pests [29], stored-product pests [30–32], and thrips [33–35] has been assessed since its introduction. By comparing with spinosad, spinetoram has been found to be more effective [25, 26, 36] against lots of important insect pests. It was also confirmed that the ecotoxicity, the mammalian toxicity and the environmental fate characteristics are as low as those of spinosad [37]. Additionally, it is safer for honeybees and bumblebees [38, 39]. More importantly, the negative cross-resistance between the Cry1Ac toxin and spinetoram may enhance the efficacy of insect pest control as well as to delay Bt resistance development [40]. Therefore, the high and selective insecticidal activity of spinetoram promotes it to be used to control cotton bollworm as a biorational insecticide, even controlling the Bt resistant cotton bollworm. In practice, for better management of insect pests, not only the direct mortality induced by insecticides but also the sublethal effects of insecticides should be considered. The sublethal effects of insecticides lead to the physiological impairments of the survivors, which manifested a series of different life phenomena, including prolonged the developmental period of surviving larvae and adults, reduced larvae’s and adults’ weight, reduced pupation ratio fecundity and so on [18, 41–44]. Thus these sublethal effects can be integrated into pest control to reduce the overuse of insecticides. So far, the information about sublethal effects of spinetoram is limited. Here we investigated the lethal effects of spinetoram to Bt susceptible and resistant cotton bollworm, and further assessed the sublethal effects of spinetoram at the LC_8_ and LC_20_ levels on cotton bollworm.

## Results

### Acute toxicity of spinetoram to Cry1Ac-susceptible and -resistant larvae

As the concentrations of spinetoram increased, mortalities of larvae were correspondingly increased after 24 h and after 72 h of exposure. In the control group, all the insects were alive. The LC_50_ values of the 96S strain arrived at 1.30 mg/kg and 0.84 mg/kg at 24 h and 72 h, respectively (Table 1). For 96-1Ac strain, the LC_50_ values were 0.62 mg/kg and 0.39 mg/kg at 24 h and 72 h, respectively (Table 1). The data here indicated that negative cross-resistance existed between spinetoram and the Cry1Ac toxin (Table 1).

**Table 1:** Acute toxicity of spinetoram against the larvae of *Helicoverpa armigera*.

Strains	Hours after treatment	Slope ± SE	LC50 (95% FL)^a^	RR^b^
96S	24	1.61 ± 0.18	1.30 (0.72–3.55)	1
BtR	24	2.79 ± 0.21	0.62 (0.44–0.92)	0.48
96S	72	3.13 ± 0.24	0.84 (0.74–0.95)	1
BtR	72	2.97 ± 0.24	0.39 (0.32–0.46)	0.46

^a^Concentration killing 50% with 95% fiducial limits in parentheses, units are mg spinetoram per kg diet.

^b^Resistance ratio, the LC_50_ for BtR strain divided by the LC50 for 96S in the same time.

### Sublethal effects on Cry1Ac-susceptible larvae

**Fig 1:**
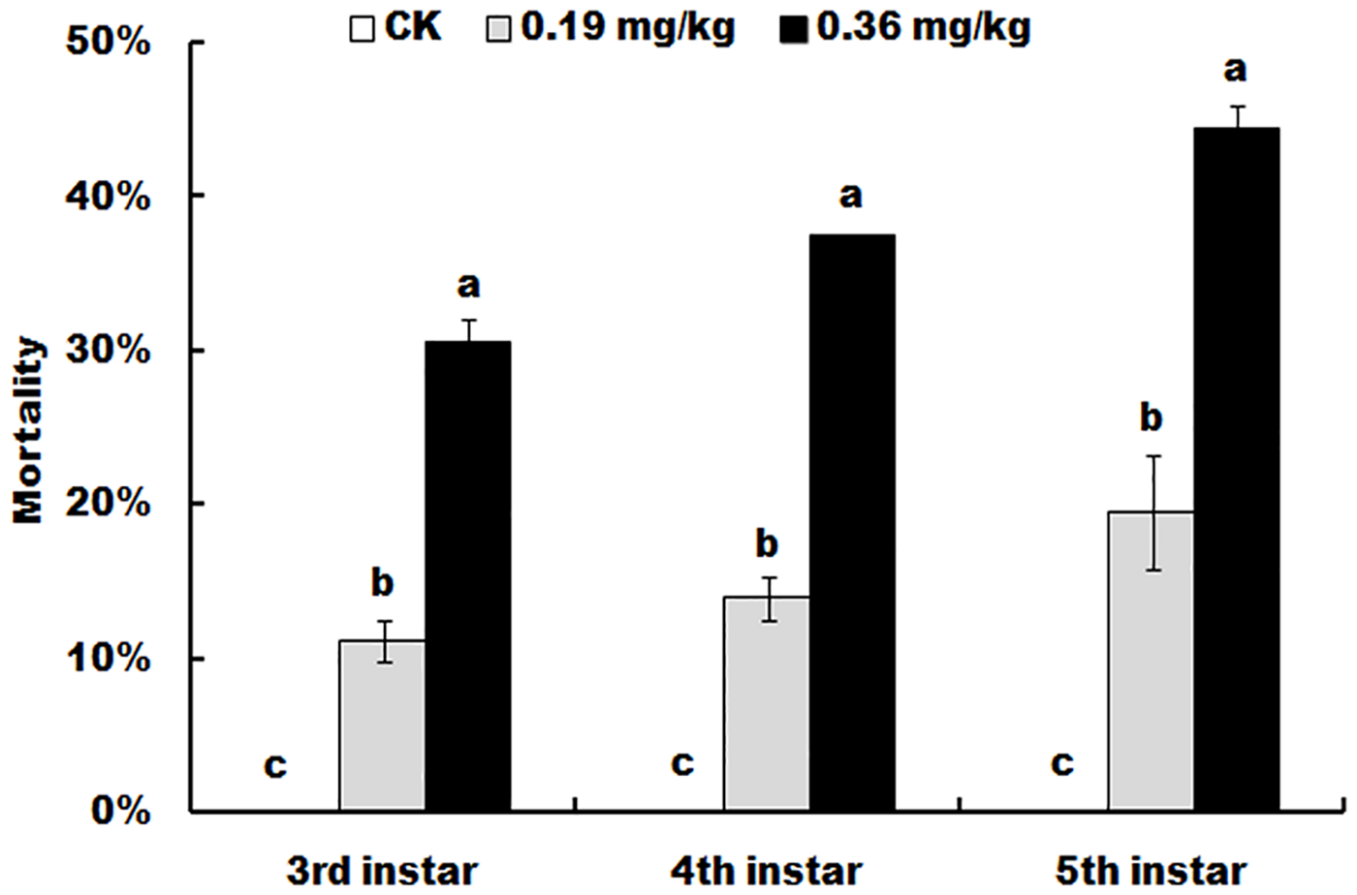
Mortality of *Helicoverpa armigera* in different larval stages when larvae were provided with diets containing spinetoram at 0 (control, ck), 0.19 or 0.36 mg/kg beginning at the late second instar. Values shown are the means and standard deviations of three separate experiments. Different letters indicate significant differences between treatments (P < 0.05, HSD test, DPS7.05).

Although spinetoram at the doses of 0.19 mg/kg (LC_8_) and 0.36 mg/kg (LC_20_) caused a relatively low fatality rate of cotton bollworm, the mortality in these two spinetoram treatments was significantly higher than that in the non-spinetoram treatment control (3rd instar: F = 186; df = 8; *P* < 0.0001; 4th instar: F = 559; df = 8; *P* < 0.0001; 5th instar: F = 96.5; df = 8; *P* < 0.0001). Mortality increased with the increases of spinetoram concentrations and contact time (Fig 1).

**Fig 2:**
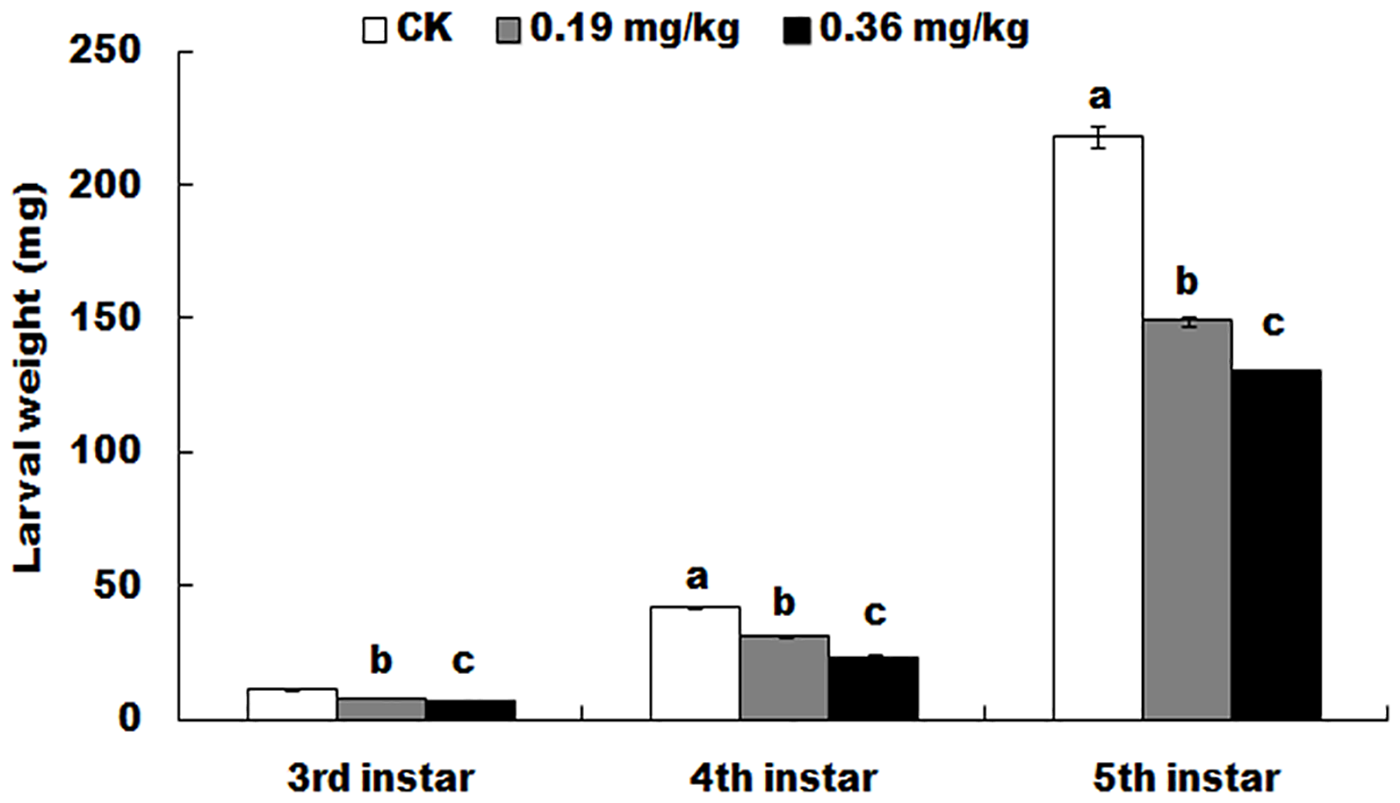
Larval wet weight of surviving *Helicoverpa armigera* exposed to different doses of spinetoram at the late second instar. Values are the means and standard deviations of three separate experiments. Different letters indicate significant differences between treatments (P < 0.05, HSD test, DPS7.05).

A negative correlation existed between the doses of spinetoram and the wet weight of larvae. The wet weight of larvae obviously decreased with the increase of spinetoram dose in the diet (Fig 2). The largest weight differences were found at the 5th instar (F = 293.9; df = 8; *P* < 0.0001), followed by the 4th-instar (F = 153.9; df = 8; *P* < 0.0001), and the smallest weight differences were observed at the 3rd instar (F = 901; df = 8; *P* < 0.0001).

**Fig 3:**
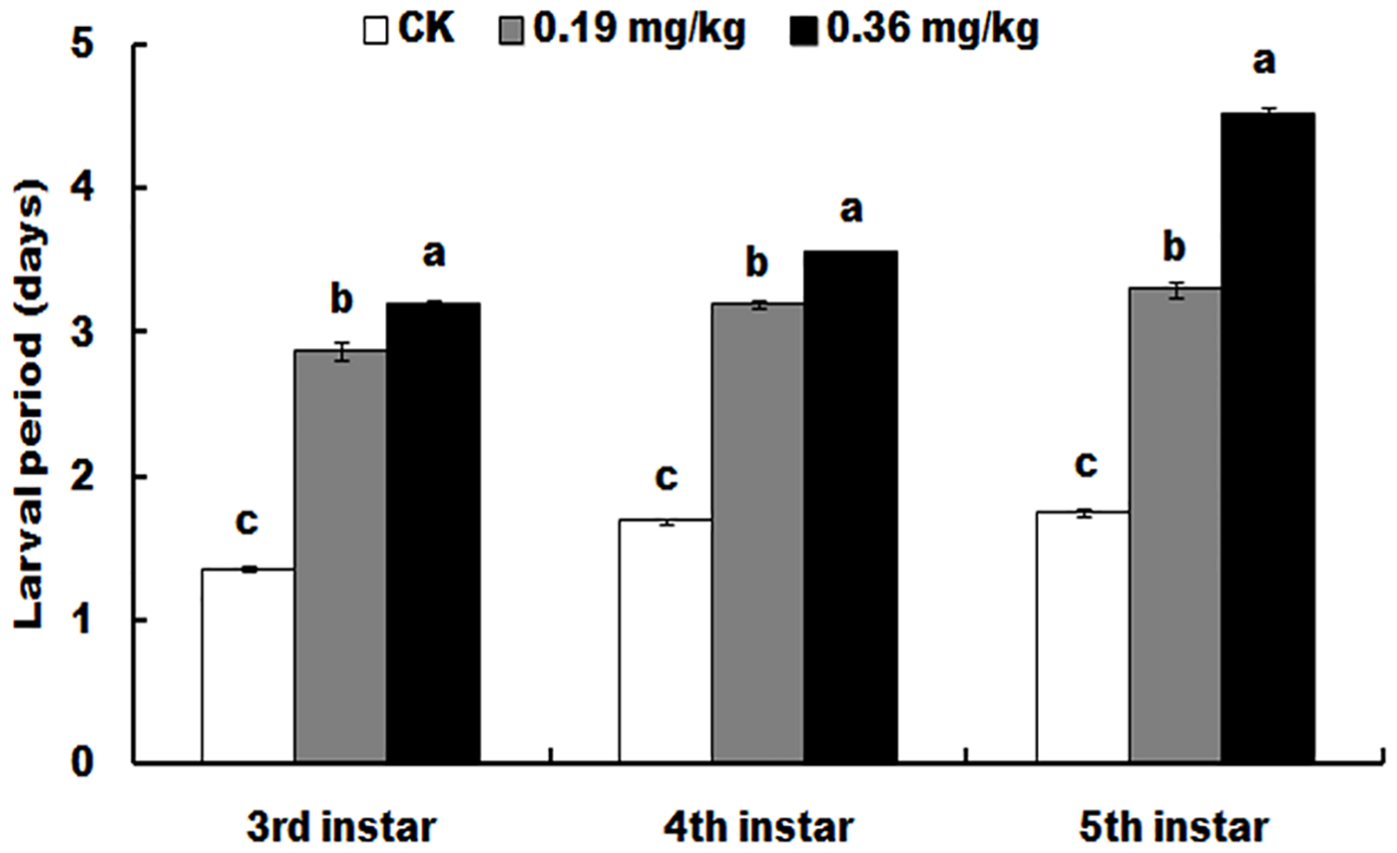
Developmental duration of each instar of surviving *Helicoverpa armigera* exposed to different doses of spinetoram at the late second instar. Values shown are the means and standard deviations of three separate experiments. Different letters indicate significant differences between treatments (P < 0.05, HSD test, DPS7.05).

The development period of each developmental stage of the larvae showed significantly prolonged among the different treatments (3rd instar: F = 713.1; df = 8; *P* < 0.0001; 4th instar: F = 1868; df = 8; *P* < 0.0001; 5th instar: F = 1180; df = 8; *P* < 0.0001). Developmental time was positively related to the concentration of spinetoram (Fig 3).

### Post-exposure effects on pupae

**Fig 4:**
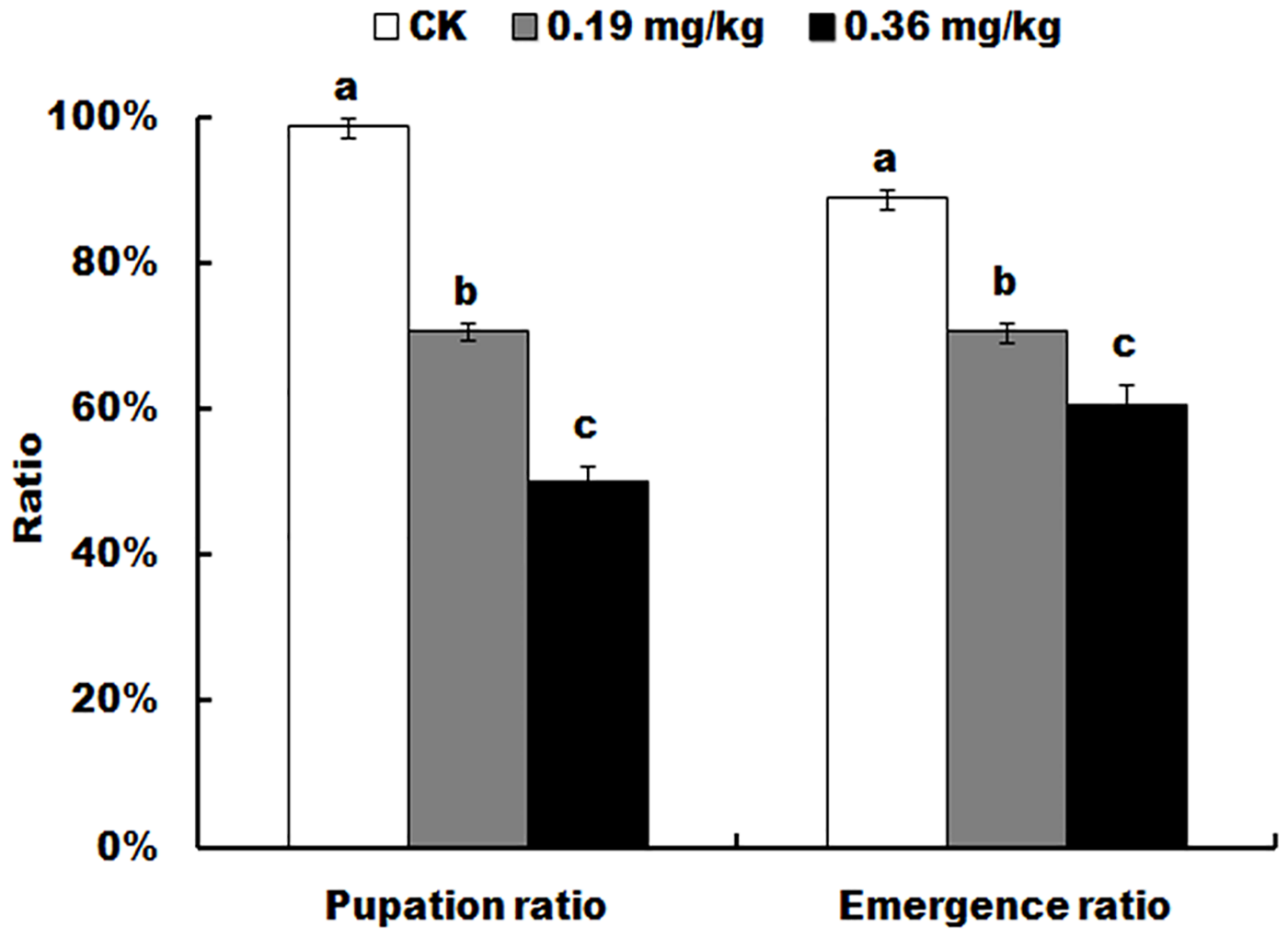
Post-exposure effects of spinetoram on the pupation and adult emergence of *Helicoverpa armigera*. Values are the means and standard deviations of three separate experiments. Different letters indicate significant differences between treatments (P < 0.05, HSD test, DPS7.05).

The survivors, which were constantly exposed to spinetoram from late second-instar, showed the post-exposure effects on pupae. Spinetoram treatment led to significant decrease of the pupation ratio and pupal survival (Fig 4). The pupation ratio of the surviving larvae showed significantly different among the three treated groups (F = 217.7; df = 8; *P* < 0.0001). With the increase of spinetoram doses, the pupation ratio of the surviving larvae significant decreased (Fig 4). Also, the adult emergence ratio (F = 43.8; df = 8; *P* = 0.003) of the surviving pupae also showed significantly different among the three treated groups, which was significantly decreased from 88.71% (control) to 70.60% (0.19 mg/kg) and to 60.32% (0.36 mg/kg).

In addition, spinetoram treatments also significantly delayed the development of prepupa (F = 634.1; df = 128; *P* < 0.0001) and pupa (F = 23.7; df = 131; *P* < 0.0001) of surviving cotton bollworm, which was pretreated with spinetoram from late second-instar (Table 2). Correspondingly, spinetoram treatment also resulted in significant decrease of the pupal weights (F = 41.1; df = 131; *P* < 0.0001) (Table 2).

**Table 2:** Effects of spinetoram on pupae of *Helicoverpa armigera*.

	Prepupal period (d)	Pupal weight (mg)	Pupal period (d)
Control	2.22 ± 0.02a	242.01 ± 5.36a	10.58 ± 0.04a
0.19 mg kg^-1^	4.43 ± 0.04b	224.56 ± 1.59b	11.72 ± 0.15b
0.36 mg kg^-1^	4.78 ± 0.03c	220.93 ± 0.61c	12.21 ± 0.15c

Results are means ± standard error (SE) of three separate experiments. Means in the same column followed by different letters differ significantly (P < 0.05) on the basis of HSD test.

### Post-exposure effects on adults and eggs

The survivors, which were constantly exposed to spinetoram from late second-instar, showed the post-exposure effects on adults and eggs. Spinetoram exposure shorten the longevity of adults, although no significant difference appeared between control and spinetoram treatments (F = 0.25; df = 103; *P* = 0.25) (Table 3). Spinetoram exposure treatment caused significant reduction in female fecundity (from 908 to 495 eggs per female adult), which was dose dependent (F = 29.4; df = 14; *P* < 0.0001).

**Table 3:** Effects of spinetoram on adult longevity, fecundity and fertility of *Herlicoverpa armigera*.

	Adult longevity (d)	Number of eggs laid per female (n)	Hatch ratio of eggs (%)
Control	11.08 ± 0.27a	908 ± 40a	93.19 ± 0.84a
0.19 mg kg^-1^	10.92 ± 0.09a	656 ± 47b	44.77 ± 3.58b
0.36 mg kg^-1^	11.00 ± 0.14a	495 ± 26c	47.10 ± 4.59b

Results are means ± standard error (SE) of three separate experiments. Means in the same column followed by different letters differ significantly (P < 0.05) on the basis of the HSD test.

Besides, the ratio of hatching eggs was greatly reduced (from 93.19% to 44.77%) at both sublethal doses of spinetoram (Table 2) (F = 64.6; df = 14; *P* < 0.0001). However, there was no significant difference between two spinetoram treatments (Table 2).

## Discussion

Based on LC_50_ values (Table 1), spinetoram showed excellent activity against cotton bollworm, especially, Cry1Ac-resistant strain. This was also confirmed by our previous data that the baseline susceptibility of cotton bollworm collected from sixteen regions in seven provinces (Hebei, Shandong, Hunan, Jiangsu, Hubei, Anhui and Henan), which showed that the spinetoram was highly toxic to cotton bollworm in the field [45]. These results were also consistent with the high toxicity of spinetoram to Lepidoptera pests previously reported [29, 46].

96-Cry1Ac strain was reported to show 1000-fold resistance to Cry1Ac [47], however showed only 0.5-fold resistance to spinetoram in the present study (Table 1). This little negative cross-resistance was consistent with the results from laboratory selection for resistance to Cry1Ac, 1400-fold resistance to Cry1Ac increased susceptibility by 2.6-fold to spinetoram in the LF60 strain [40, 48]. More importantly, these field populations of cotton bollworm collected from above mentioned regions have shown small but significant increases in resistance to Bt in China [14–16, 49], however showed more susceptible to spinetoram (Table 1) [45]. Especially, it was confirmed that the LC_50_ of spinetoram (0.19 mg kg^-1^) was the lowest to the cotton bollworm from Xiajin among these sixteen regions based on toxicology testing of first-instar larvae [45]. However these cotton bollworm from Xiajin showed 7.7-fold resistance to Bt cotton in 2011 [49] and about 1.6-fold resistance to Bt cotton in 2013 [16]. These results altogether revealed that the resistance to Bt (no matter higher or lower Cry1Ac-resistance level) can lead to more susceptible of Cry1Ac-resistance *H*. *armigera* to spinetoram. The current results suggest that spinetoram is a good choice against *H*. *armigera*, especially, it can be used to control the Bt-resistance strains of *H*. *armigera*. Unfortunately, this kind of the negative cross-resistance mechanism is surprisingly limited so far. Although the resistance to Cry1Ac in LF60 was tightly linked with a mutant allele that disrupted the ABC transporter protein ABCC2 [48], the increased susceptibility to spinetoram in LF60 is not genetically linked with the same mutation [40]. More studies should be performed on to address the negative cross-resistance mechanism in future.

Because most of the field strains of cotton bollworm showed susceptible or lower resistance to Bt cotton in China [50], this study also revealed many sublethal effects of spinetoram on Bt susceptible cotton bollworm. The sublethal doses of spinetoram can suppress weight gain of larvae, delay larval and adult development and prolong the prepupal and pupal periods (Figs 2 and 3, Tables 2 and 3). Similar physiological phenomena were found in other spinosyns treatments previously reported [27], like spinosad [18]. Since spinetoram belongs to spinosyns, it is not strange that these pesticides can cause similar physiological phenomena of larvae [18]. Importantly, the above sublethal effects of spinetoram, as well as other spinosyns may cause the changes of occurrence date and occurrence period of cotton bollworm.

Besides the direct effects of spinetoram to the larvae, post-exposure effects of spinetoram on cotton bollworm were also observed. Spinetoram at the sublethal doses significantly reduced the pupation ratio, pupal survival, female fecundity and the ratio of hatching eggs. Similarly, in *Plutella xylostella* and *H*. *armigera*, spinosad at lethal or sublethal doses caused significant decrease of the fecundity [18, 41]. Even the spinetoram at low concentrations in grain can also suppress progeny production of *R*. *dominica* and *P*. *truncatus* [32]. Also, for *S*. *granaries*, progeny production was also significantly suppressed by spinetoram [51]. In contrast, for *Tetranychus urticae*, spinetoram at LC_10_ and LC_20_ doses reduced the developmental time from egg to adult and increased the fecundity [52]. In addation, for beneficial insects, including honey bees and bumblebees, the sublethal effects of spinetoram did not change their foraging behavior [38, 39]. The discrepancies of the effects of spinetoram on different insect species indicated that spinetoram may have the characteristics of specificity and selectivity. Otherwise, for cotton bollworm, the effects of spinetoram, such as the reduction in the survival rate of larvae, pupation ratio, female fecundity and the ratio of hatching eggs, indicated that it can suppress the cotton bollworm density of the next generation.

In summary, the present results suggest that not only the lethal effects but also the sublethal effects of spinetoram could have a negative influence on the dynamics of cotton bollworm. Moreover, the susceptibility of *H*. *armigera* (Hebei, Shandong, Hunan, Jiangsu, Hubei, Anhui and Henan) to spinetoram [45] and the negative cross-resistance between Cry1Ac and spinetoram [40] make spinetoram a good choice for suppressing the recovery of *H*. *armigera*. So, we suggested spinetoram can be used as a biorational insecticide in pratical integrated pest management (IPM) programs for better management of cotton bollworm. It’s also worth noting that both the lethal effects and sublethal effects of spinetoram should be taken into consideration when pest control strategies are made.

## Materials and methods

### Insect

In this study, 96S *H*. *armigera* was selected as a susceptible strain, that was collected from Xinxiang County (Henan Province, China) in 1996 and cultured on an non-insecticide artificial diet [53]. The 96-1Ac strain was selected with a solubilized Cry1Ac protoxin for the first 60 generations [53] and with MVPII in subsequent generations [47]. This 96-1Ac strain had about 1000-fold resistance to Cry1Ac protoxin in the latest reports [47]. All the above insects were reared in the laboratory under the environment of 75±10% RH, 27±1 °C and a photoperiod of 14:10 (L:D) h [53].

### Acute toxicity assays

85.8% spinetoram was kindly supplied by Jun Ning from State Key Laboratory for Biology of Plant Diseases and Insect Pests, Institute of Plant Protection Chinese Academy of Agricultural Science. Larval mortality was evaluated for a range of spinetoram concentrations from 0 to 3.2 mg/kg diet after 24 h and 72 h of exposure. The concentrations of spinetoram were progressively diluted by water and then mixed with artificial diet. For assessing the acute toxicity of spinetoram to 96S and 96-1Ac strains, the uniform size late second instar larvae were used for the bioassay. For each treatment, totally 72 individuals was tested. After exposing to the spinetoram 24 h and 72 h, mortality was recorded based on the same criterion that insects did not respond to the stimulation with a brush were adjudged to be dead [18].

### Sublethal effects of spinetoram

For assessing the sublethal effects of spinetoram, we employed two spinetoram treatments at sublethal concentrations and a spinetoram-free (water) control in this study. These two sublethal concentrations were 8% [(LC_8_) = 0.19 mg kg^-1^] and 20% lethal concentration [(LC_20_) = 0.36 mg kg^-1^], which were selected based on the baseline toxicity of spinetoram to 96S strain at 24h. The same size late second-instar larvae were selected to conduct this assessment. The fresh artificial diet (containing the sublethal doses of spinetoram or water) was replaced every two days. The survival of each treatment and the growth of each individual were recorded every two days until adult emergence. Moreover, we also observed the duration of each larval instar, the development periods of the prepupal and pupal stages, the wet weight of larvae at the first-day of each instar, the pupal weight, the ratio of pupation and adult emergence.

The fecundity was evaluated by putting surviving moths in a 40 × 40 cm cage to mate for approximately 2 days after emergence under the condition of artificial feeding in the laboratory [18]. Then, we transferred the mating pairs to small cups and covered the cups with gauze. The moths also reared under the same laboratory conditions as the above description. Every day, we the changed fresh gauze for each cup, and counted the numbers of eggs on the gauze until all females died. Meanwhile, the longevity of adult moths was also recorded for each treatment. For the ratio of hatching eggs study, we randomly taken fifty eggs from each pair of adult moths, and recorded the numbers of hatching eggs.

All the above experiments were done three replicates, and at least 60 individuals per replicate. The criterion for judging live larvae and adults was that they can crawl when stimulated with a fine-haired brush. And the judge criterion for the live pupae was they can successfully moult to moths [18].

### Statistical analysis

LC_50_ values for spinetoram were estimated by probit analysis. One-way analysis of variance (ANOVA) was used to statistically analyze the above data, the significantly differences among the three treatments were compared with Tukey’s honestly significance difference (HSD) test. All the above data analyses were performed with DPS7.05 at the P < 0.05 level of significance.
